# Improving the organization and interactivity of metabolic pathfinding with precomputed pathways

**DOI:** 10.1186/s12859-019-3328-x

**Published:** 2020-01-10

**Authors:** Sarah M. Kim, Matthew I. Peña, Mark Moll, George N. Bennett, Lydia E. Kavraki

**Affiliations:** 1 Department of Computer Science, Rice University, Houston, Texas USA; 2 Department of BioSciences, Rice University, Houston, Texas USA

**Keywords:** Metabolic pathfinding, Precomputation, Atom mapping, Graph search

## Abstract

**Background:**

The rapid growth of available knowledge on metabolic processes across thousands of species continues to expand the possibilities of producing chemicals by combining pathways found in different species. Several computational search algorithms have been developed for automating the identification of possible heterologous pathways; however, these searches may return thousands of pathway results. Although the large number of results are in part due to the large number of possible compounds and reactions, a subset of core reaction modules is repeatedly observed in pathway results across multiple searches, suggesting that some subpaths between common compounds were more consistently explored than others.To reduce the resources spent on searching the same metabolic space, a new meta-algorithm for metabolic pathfinding, Hub Pathway search with Atom Tracking (HPAT), was developed to take advantage of a precomputed network of subpath modules. To investigate the efficacy of this method, we created a table describing a network of common hub metabolites and how they are biochemically connected and only offloaded searches to and from this hub network onto an interactive webserver capable of visualizing the resulting pathways.

**Results:**

A test set of nineteen known pathways taken from literature and metabolic databases were used to evaluate if HPAT was capable of identifying known pathways. HPAT found the exact pathway for eleven of the nineteen test cases using a diverse set of precomputed subpaths, whereas a comparable pathfinding search algorithm that does not use precomputed subpaths found only seven of the nineteen test cases. The capability of HPAT to find novel pathways was demonstrated by its ability to identify novel 3-hydroxypropanoate (3-HP) synthesis pathways. As for pathway visualization, the new interactive pathway filters enable a reduction of the number of displayed pathways from hundreds down to less than ten pathways in several test cases, illustrating their utility in reducing the amount of presented information while retaining pathways of interest.

**Conclusions:**

This work presents the first step in incorporating a precomputed subpath network into metabolic pathfinding and demonstrates how this leads to a concise, interactive visualization of pathway results. The modular nature of metabolic pathways is exploited to facilitate efficient discovery of alternate pathways.

## Background

Identifying novel metabolic pathways for synthesizing valuable products is an important first step in the field of metabolic engineering, where organisms are genetically manipulated to serve as cellular factories for producing valuable chemicals. Recent advances in the field of metabolic engineering have proven successful in optimizing the production of biofuels [1, 2], pharmaceuticals [3–6], and chemical precursors [7].

Identifying the metabolic pathways used to synthesize these products has traditionally required an expert to search through metabolic databases and literature for promising enzymatic reactions. However, the rapid expansion of available metabolic information stored across many online databases has made it increasingly difficult to manually survey all possibilities and resources. To address the need for faster and broader search, several computational approaches have been developed to identify promising pathways, resulting in the identification of novel pathways like the synthesis of 1,4-butanediol (BDO) [8], a high-demand commercial chemical precursor that had a market of one billion tons annually in 2013 [7].

One of the universal challenges of automated pathfinding methods is the trade-off between finding a set of pathways that presents diverse alternatives for synthesizing the target product, while also not overwhelming the user with too many options. Introducing strict constraints during the search can limit the diversity of pathways found, but too few constraints will result in a large number of repetitive or biologically infeasible pathways. How the results are presented and visualized also plays a significant role in this trade-off, as a means of visualization can enable the user to easily sift through a large number of results.

This paper introduces a meta-algorithm designed for metabolic pathfinding, which could be generally applied to other graph-based search approaches giving rise to a large number of unique pathways. We demonstrate that our meta-algorithm and interactive visualization can be coupled allowing for post-search filtering that provides the user with information about a manageable number of pathways instead of listing all generated pathways, which requires significant manual inspection.

The meta-algorithm proposed in this paper is built upon graph-based metabolic pathfinding algorithms, such as [9–12]. Graph-based methods find pathways based on the connectivity of compounds in a metabolic network and also lend themselves well to scaling to large metabolic networks and finding pathways that incorporate enzymes from multiple organisms. However, the flexibility of the graph-based search approach also results in finding thousands of biologically infeasible pathways. This paper contributes a new algorithm for metabolic pathfinding, which besides its good performance, facilitates interpretation of the results.

Existing graph-based search approaches have utilized a combination of heuristics and cutoffs to limit the number of infeasible pathways [13]. One of the most widely used heuristics is the conservation of chemical structure from the start to the target compound. Pathway Hunter Tool [14], MetabolicTinker [15], and GEM-Path [16] incorporate chemical similarity measures as heuristics to guide the metabolic search. Other methods track individual atoms, or groups of connected atoms in the case of AGPathFinder [17], from the start to target compound and aim to conserve a minimum number of atoms throughout the pathway [9, 12, 18] or maximize the number of atoms conserved [10, 19]. Information on enzymatic reactions, like thermodynamic favorability (*Δ*G) [15, 17, 20] and enzyme efficiency and promiscuity [20, 21], is also used to guide the search and rank pathways. It is important to note that many of these pathfinding methods use a combination of several heuristics. For example, in addition to factoring in chemical similarity and thermodynamic favorability, XTMS [22] incorporates metabolic exchange information, compound toxicity scores, and estimated maximum pathway yield in ranking pathways. However, even when using these heuristics, pathfinding searches can return thousands of results, which are either listed sequentially in full or limited by a cutoff of the top x pathways. Though users can adjust the search by changing input parameters (i.e., changing the minimum number of atoms to conserved), user interaction with the results themselves is limited.

Across existing pathfinding methods, pathway results are most commonly presented in a ranked list [9, 19], which requires users to evaluate each pathway result individually. Instead of a list, Metabolic Tinker [15] presents an overview of pathways by providing a static output graph that represents a combination of all pathway results. Pathway results that share all the same compound intermediates are grouped together and considered the same pathway. However, this organization does not help group similar pathways together if the pathways do not go through the same compound intermediates. Also, the graph produced by Metabolic Tinker is static and does not come with any tools for users to alter or explore the output graph after the search has been completed. For more general exploration of the metabolic space around a target compound, both BioSynther [23] and ATLAS [24] provide views that display all compounds that are a given number of reaction steps away from a given compound. However, this visualization becomes exponentially crowded with increasing step size, considering there are no ways for the user to filter pathways. Other tools incorporate user interaction with exploration of pathways. The MRSD tool designed by Xia et al. [21] and BioSynther [23] provide views where the user can specify a compound, then interactively select intermediate compounds step by step, manually constructing a pathway. This works well for finding small variants of a pathway of interest or if the user has a specific pathway in mind. However, in the case where the user is more interested in exploring all possible pathways for synthesizing a target compound, the interactive step-by-step approach to exploring possible pathways can be time consuming and may not provide a good overview of all the possibilities, especially if there are many diverse routes to explore.

One aspect that is not addressed by previous search methods is the identification of common segments across different search results. Many pathways of interest in metabolic engineering involve synthesis of a more complex target compound (e.g., artemisinin) from relatively simple compounds like glucose. In several of these synthesis pathways, simple compounds must first be converted into common intermediate precursor compounds before being synthesized into the final target compound. The modularity of metabolic networks has been observed by previous work [25–28], and common reaction modules across known pathways have even been identified by [29]. However, these reaction modules are based on existing natural pathways and do not necessarily generalize to the heterologous pathways found by metabolic pathfinding algorithms. We have observed that the subpaths between common precursor compounds are often shared across several different heterologous pathways, acting as modules that repeatedly appear across pathways. Given that these common subpath modules can be identified without extensive expert knowledge, precomputing these modules to look up during the search can keep metabolic pathfinding algorithms from having to re-explore the same metabolic space in future searches and aid with condensing the visualization of the results.

### Contributions

This paper introduces and demonstrates an initial approach to take advantage of short series of connecting reactions among common compounds across metabolic pathways by incorporating precomputed subpaths into graph-based metabolic pathfinding search algorithms. To our knowledge, no previous approaches have incorporated the use of precomputed pathways. In addition to their use as look-up modules in pathway searches, precomputed pathways can help to organize and quickly visualize pathway results, enabling a more interactive user experience. Though interactive graph visualization of known pathways has been explored [30–32] and included in recent pathway tools like PyPathway [33], this visualization has not yet been applied to metabolic pathfinding results. This paper also introduces the use of post-search sliding filters which enable the user to evaluate the validity of result pathways based on path length, carbons conserved, and ATP used. The introduction of post-search filters provide a more interactive experience for users that has not been provided by previous metabolic pathfinding methods, enabling users to select pathways based on their own interests and encouraging broader exploration of the search results without overwhelming the user.

## Results

**Fig. 1:**
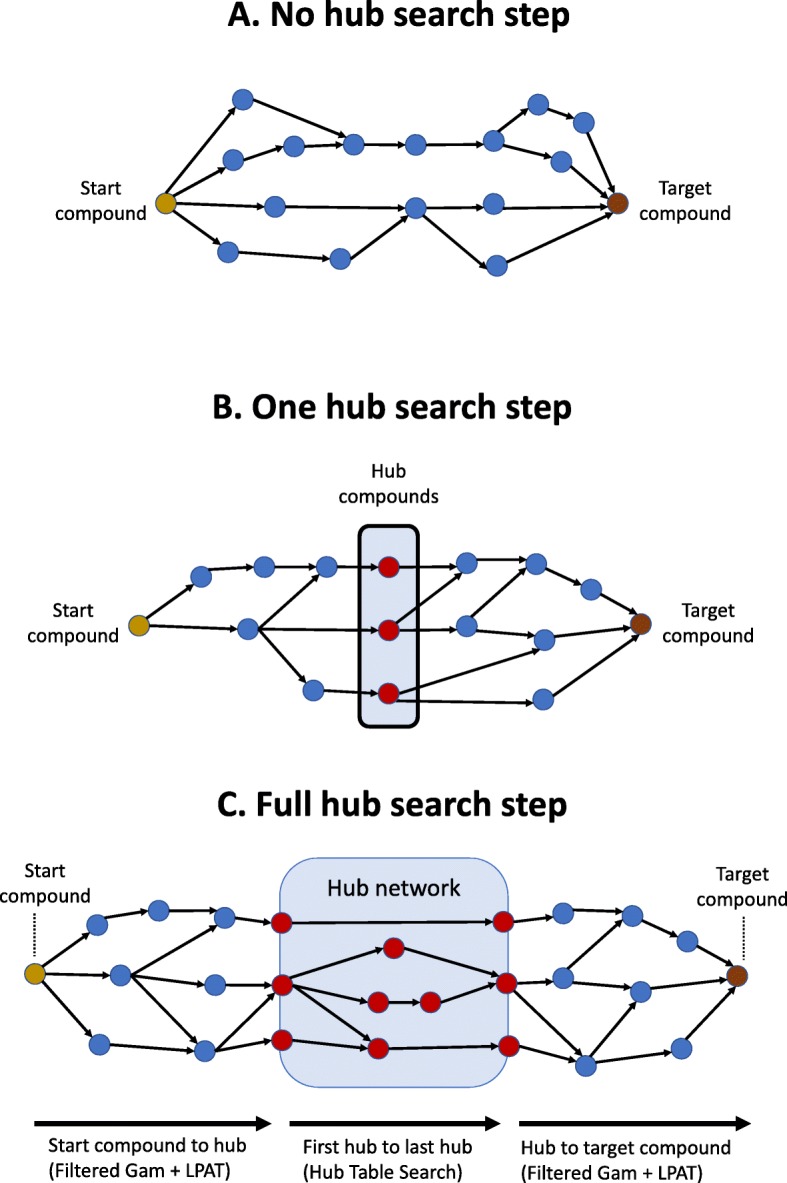
Overview of hub search process. Pathways with no hubs (**a**) and one hub (**b**) are found using an existing linear atom-tracking graph-based pathway search (LPAT [9]) while excluding hub compounds from the metabolic network. The process for finding pathways with at least two hubs (**c**) consists of (1) the start compound to “first” hub compounds search, (2) the “first” hubs to the “last” hubs search using the precomputed network, and (3) the “last” hubs to the target compound search. The “first” hubs and “last” hubs are identified by using a chemical similarity heuristic (SIMCOMP [50]) to find the hub compounds that have the most similar chemical structures to the start compound and target compound respectively. The first and last hub compounds are identified before the hub to hub search is performed. For all pathways identified by the hub pathway search, at least two carbon atoms are conserved from the start compound to the target compound to avoid finding pathways that do not utilize the start compound to synthesize the target compound

The Hub Pathway Search with Atom Tracking (HPAT) was developed as a first step to utilizing precomputed subpaths in the metabolic pathfinding search (see Fig. 1 for a visual overview of the HPAT meta-algorithm). First, all subpaths between a set of highly connected hub compounds are precomputed and stored in a look-up table.

Then, given a start compound and a target compound, HPAT finds pathways converting the start to the target compound in three search steps: (1) a no-hub pathway search, where all hub compounds are excluded from the searched metabolic network, (2) a one hub pathway search, where all but one hub compound are excluded, and (3) the hub pathway search, which utilizes the precomputed subpaths.

In this study, the linear atom-tracking search algorithm LPAT [9] was used to precompute subpaths between hub compounds, find pathways containing one or less hub compounds (Fig. 1a-b), and find the segments of the hub pathways connecting the start compound to the first hub compound and the last hub compound to the target compound (Fig. 1c). Only the hub compounds that have the most similar chemical structures to the start and target compounds are included in the search as the first and last hub compounds respectively (See the “First and last hubs selection” subsection in “Methods” section for more details).

Throughout each step of the hub pathway search, carbon atoms are tracked from the start compound to the target compound to insure that carbon atoms from the start compound are being incorporated into the target compound. Conserving carbon atoms throughout the pathway prevents the search from finding infeasible pathways that go through currency metabolites that only contribute energy or electron balance to the reaction.

A hub pathway search tool will ideally find biochemically feasible pathways that do not already occur naturally in existing organisms. Thus the performance of the HPAT meta-algorithm was evaluated based on its ability (1) to find a diverse set of known biochemically feasible pathways documented in the literature, and (2) to find novel pathways that do not occur in existing organisms and have the potential to be used for metabolic engineering applications.

This section then describes how pathways found by HPAT are visualized on the Hub Pathways Webserver, and how users can utilize interactive filtering options to selectively reduce the number of results displayed.

### Recovery of Known Pathways by HPAT

The quality of pathways found by HPAT were evaluated by comparing the HPAT results to nineteen known pathways taken from the literature and KEGG database [34, 35]. The final products of the pathways were chosen because of their importance as precursors to the synthesis of industrial or medicinal compounds. The start and target compounds of the test set pathways varied in size and complexity. The smallest compounds (pyruvate, 3-hydroxypropanoate, and 1,3-propanediol) only contained three carbon atoms, whereas the largest compound (stachyose) contained twenty four carbon atoms and several ring structures (See Additional file 1). The test set pathways also varied in path length, ranging from four to fifteen enzymatic reactions. Five variants of the lysine synthesis pathway and seven variants of the 3-hydroxypropanoate (3-HP) synthesis pathway were also included in the known pathway set to test if HPAT’s could find a diverse range of results.

To evaluate the HPAT meta-algorithm’s ability to find the known pathways, the F_1_ score [36] was calculated for each result pathway based on its reactions. Reactions in the result pathway that are also present in the known pathway are counted as true positives (TP), whereas reactions in the result pathway that are not present in the known pathway are counted as false positives (FP). Reactions that are in the known pathway but not the result pathway are counted as false negatives (FN). The F_1_ score is calculated as F_1_ = 2 ×(P ×R)/(P+R), where P = TP/(TP+FP) is the precision and R = TP/(TP+FN) is the recall.

The highest F_1_ scores of all pathways found by HPAT compared to each known pathway is reported in Table 1. This differs from previous studies, where only the top handful of pathways, determined by ranking heuristics like path length and thermodynamics, are compared to the known pathway. However, unlike previous studies, it is assumed that the user of the HPAT search can utilize interactive post-search filtering tools to narrow down the results to a manageable set of pathways, such that the ranking of the results does not have a significant impact on which pathways the user sees. For sake of reference, the F_1_ scores were also calculated for the closest pathway (1) among the top twenty pathway results and (2) among all the results obtained from a LPAT search using comparable input parameters (see Additional file 5 for values of LPAT input parameters). The F_1_ scores were calculated for the top twenty LPAT results to evaluate if a user could quickly find the canonical pathway in the top few LPAT results without utilizing the interactive filtering tools.

**Table 1:** F_1_ scores of the closest pathway found by the HPAT meta-algorithm (uses precomputed subpaths) and LPAT algorithm (does not use precomputed subpaths) compared to known pathways, where an F_1_ score of one indicates that the HPAT search found the exact known pathway

		F _***1***_ Score, LPAT	F _***1***_ Score, LPAT
Known pathway	F _***1***_ Score, HPAT	(Top 20 Paths)	(All Paths)
Pyruvate → Lysine I	1.00	0.70	0.70
Pyruvate → Lysine II	1.00	0.70	0.70
Pyruvate → Lysine III	1.00	1.00	1.00
Pyruvate → Lysine IV	1.00	0.78	0.78
Pyruvate → Lysine V	1.00	0	0
Glutamate → Proline	1.00	1.00	1.00
UDP-Galactose → Stachyose	1.00	1.00	1.00
alpha-D-glucose → Phenylpyruvate	0.71 ^+^	0.71	0.71
alpha-D-glucose → Dopamine	0.59 ^*†*^	0.42	0.76
*Pyruvate → 3-HP, I	1.00	1.00	1.00
*Pyruvate → 3-HP, II	0.50 ^*†*^	0.33	0.33
*Pyruvate → 3-HP, III	0.60 ^*†*^	0.38	0.78
*Pyruvate → 3-HP, IV	0.42 ^*†*^	0.71	0.71
*Pyruvate → 3-HP, V	0.36 ^*†*^	0.40	0.40
*Pyruvate → 3-HP, VI	0.36 ^*#*^	0.36	0.36
*Pyruvate → 3-HP, VII	0.53 ^*#*^	0.36	0.50
Glucose → Glucaric acid	1.00	1.00	1.00
alpha-D-glucose → 1,3-propanediol	1.00	0.67	1.00
Tryptophan → Melatonin	1.00	1.00	1.00

The closer the F_1_ score is to one, the more similar the found pathway’s reactions are to that of the known pathway. The first column of F_1_ scores for LPAT is calculated based on the closest pathway within the top twenty pathways found by LPAT, while the second column of F_1_ scores for LPAT is calculated based on the closest pathway for all LPAT pathway results

^*^Pathway is experimentally validated; does not exist naturally in organisms

^#^Requires an engineered, non-natural 2,3-alanine aminomutase

^+^Known pathway conserves less than two carbons

^*†*^Hub in canonical pathway closest to start/target not among closest hubs

The HPAT meta-algorithm was able to find the exact canonical pathway for eleven of the nineteen test cases, including all five variants of the lysine synthesis pathway. Meanwhile, the LPAT algorithm was able to find the exact canonical pathway for only seven of the nineteen test cases and found only one of the five canonical lysine synthesis pathways. The reason HPAT could outperform LPAT in these cases is that the maximum search depth used for finding the precomputed hub paths (100,000) was larger than the depth used for the HPAT hub search and LPAT search (10,000, see Additional files 3, 4 and 5). This enables more diverse subpaths to be precomputed and then later looked-up during the HPAT search.

Evaluating just the top twenty LPAT results (instead of all LPAT results) yielded lower F_1_ scores for four of the pathway test cases (dopamine, 3-HP III, 3-HP VII, and 1,3-propanediol). Though the known 1,3-propanediol synthesis pathway was found by LPAT, it was not contained in the top twenty LPAT results. This example illustrates how HPAT with interactive filtering can often enable users to find known pathways as accurately as LPAT without having to look through as much information.

For eight of the nineteen test cases, the HPAT meta-algorithm did not find the known pathway. There are three main reasons for this: (1) the known pathway requires an engineered, non-natural enzyme, (2) the known pathway does not conserve at least two carbons, and (2) the first or last hub compound(s) in the known pathway were not identified as the closest hubs to the start or target compound by the chemical similarity heuristic (See Table 1).

For the first case, the reaction catalyzed by the engineered enzyme in these known pathways was not present in the KEGG database, so it was not possible for the HPAT search to find these pathways. For the second case, the selected input parameters for both the HPAT and LPAT searches impose the restriction that all result pathways must conserve a minimum of two carbons atoms from the start to the target compound. Though conserving one carbon atom from start to target compound prevents the search from finding pathways through currency metabolites that are not used to build up the target compound, conserving a single carbon atom does not prevent the search from finding nonsensical pathways that break down the start compound to a single carbon compound (i.e., CO_2_), only to build back up to the target compound. The two carbon atom limit was chosen to reduce the number of these particular nonsensical pathways. However, if the known pathway only conserves one carbon atom from start to target compounds, the pathway will not be found by either HPAT or LPAT due to this two carbon atom limit setting. In these cases, the HPAT meta-algorithm performed about the same or slightly better than the LPAT algorithm in finding pathways similar to the known pathway.

For the third case, whenever the start or target compound are not hub compounds, the HPAT search will only explore pathways that go through the first and/or last hub compounds identified by a chemical similarity heuristic (see “Methods” section for details), so if the first or last hub compound of the known pathway is not one of the most similar compounds, the HPAT search will not find the pathway. In these cases, the HPAT meta-algorithm tended to perform worse than LPAT in finding pathways similar to the known pathway.

### Identification of novel pathways by HPAT

Biochemical repositories, such as KEGG, are curated to maintain completeness and accuracy of standard metabolic reactions, but reactions for enzymes with either engineered or off-target function are often not included. This is an inherent constraint on the discovery of novel pathways. Despite this limitation, novel approaches and useful pathways can be uncovered using exploratory tools such as the one described in this paper that can filter results to favor carbon conservation from start to target compound or utilize enzymes drawn from disparate species to form heterologous pathways. Here, we look more closely at 3-hydroxypropionic acid (3-HP), the biosynthesis of which is characterized by multiple standard and nonstandard pathways.

3-HP, a compound of interest for numerous metabolic engineering projects, has had at least seven biosynthesis pathways starting from glucose that have been patented and evaluated to determine if they are "biologically attractive," meaning the pathways are thermodynamically favorable, maintain redox balance, and require minimal ATP utilization [37–42]. Pyruvate is a sensible starting point for pathfinding the synthesis of many compounds, including 3-HP, because it is the natural product of glycolysis, a central pathway of organisms consuming glucose as a carbon source. 3-HP is a good test of the hub search because of the variety of nonstandard biosynthesis pathways that include hub compound intermediates.

In Table 1, we can see that of the seven previously defined pathways only one is recovered in its entirety independent of the selected hubs: Pathway I, which passes through lactate. Two additional pathways are recovered for specific hub sets: Pathways III and IV, which both include the carboxylation of pyruvate to oxaloacetate but differ in the conversion of *β*-alanine to 3-oxopropanoate. Of the remaining four pathways, only partial coverage is obtained either from overlap with Pathways I, III, and IV or because individual reaction steps are present in the cumulative pathfinding results but not in a reaction sequence matching the illustrated pathways. Pathways VI and VII cannot be completed because the conversion of *α*-alanine to *β*-alanine requires an engineered, non-natural 2,3-alanine aminomutase, which is absent from KEGG [38].

**Fig. 2:**
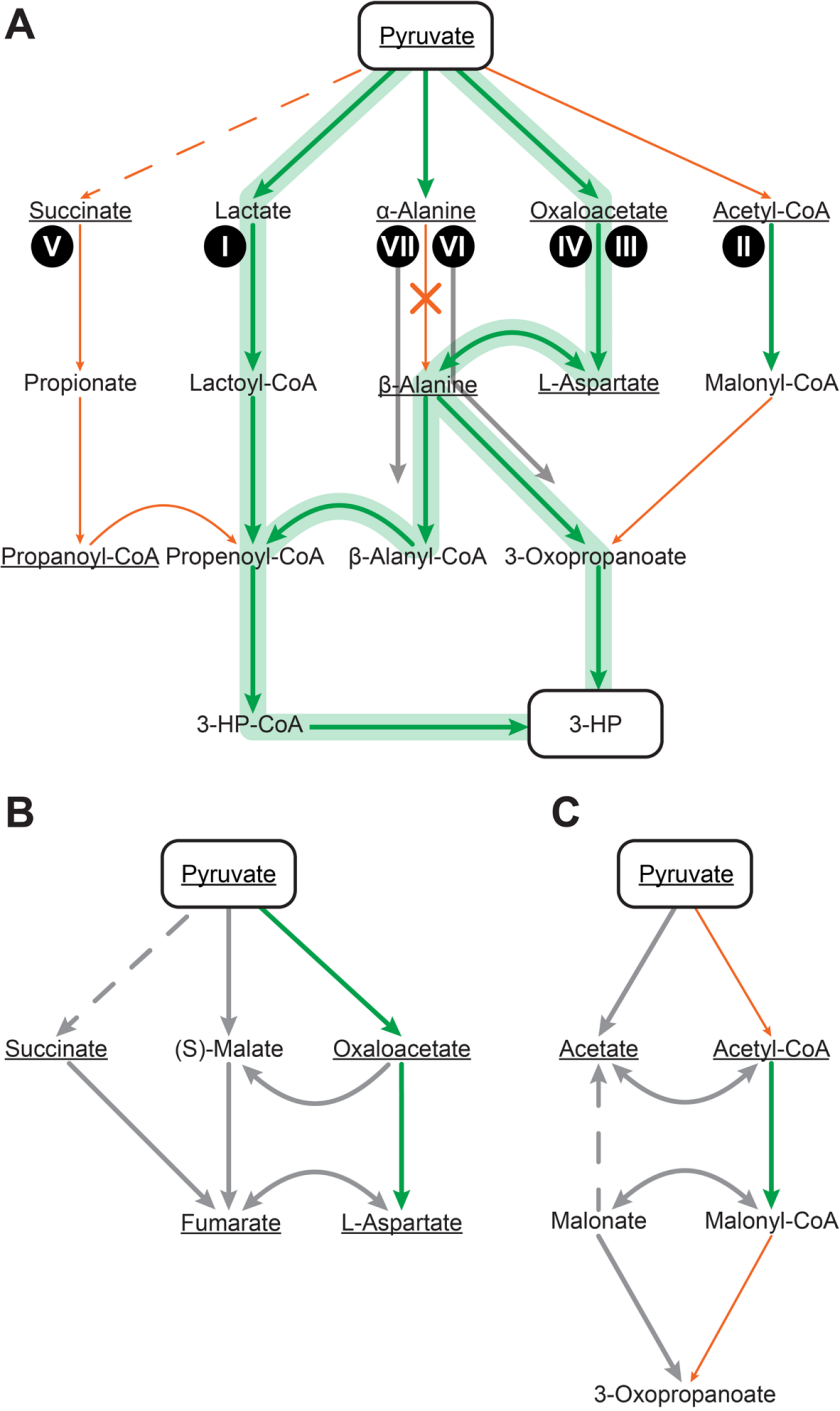
HPAT can recover three of seven pathways patented for use in the production of 3-hydroxypropionic acid (3-HP) starting from pyruvate. The seven pathways are numbered according to Jiang et al. [41]. Intermediates that are underlined represent hub compounds from the 50 in-degree (I) hub table (see Table 2 for details). Arrows represent reactions linking intermediates where the color of the arrow indicates if they are found by the HPAT search where at least one carbon is conserved. Solid green arrows represent reactions that were found by the HPAT search, solid orange arrows represent reactions that were not found in the search, dashed arrows are require multiple reactions, and an X marks those reactions that are absent from KEGG and cannot be found by the search, grey arrows represent alternative reactions found by the search but not associated with the seven pathways, and the green highlight represents entire pathways between pyruvate and 3-HP that are recovered. **a** Reactions identified in the HPAT search connecting TCA cycle intermediates, succinate, malate, and fumarate, to Pathways III and IV (**b**) and viable acetate and malonate to Pathway II (**c**)

Of the remaining pathfinding results, the majority are alternative, feasible paths that have been documented in KEGG between the illustrated intermediates. These alternative pathways are valuable because they represent points where potentially more carbons can be fed into a pathway of interest. An example of this is seen for intermediates of the TCA cycle, succinate, fumarate, and malate, which can be fed into the oxaloacetate and aspartate nodes of pathways III and IV (Fig. 2b). Similarly, we can elaborate on pathway II by adding a parallel pathway of pyruvate to acetate to malonate to 3-oxopropanoate (also referred to as malonate semialdehyde). Between these two parallel pathways, acetyl-CoA and malonyl-CoA can each be converted to acetate and malonate, respectively (Fig. 2c).

**Table 2:** Description of hub tables that were evaluated

Hub Table	Description
20 (O)	Selected twenty hub compounds involved as reactants in the most number of reactions (highest out-degree). Subset of the 50 (O) hub table.
50 (O)	Selected fifty hub compounds involved as reactants in the most number of reactions (highest out-degree). Subset of the 80 (O) hub table.
80 (O)	Selected eighty hub compounds involved as reactants in the most number of reactions (highest out-degree).
139 (A)	139 hubs used in the Araki et al. [43] study.
50 (IO)	Selected fifty hub compounds involved in the most number of reactions (either as reactants or products, highest degree).
50 (I)	Selected fifty hub compounds involved as products in the most number of reactions (highest in-degree).
50 (R1) and (R2)	Two independent sets of fifty randomly selected hub compounds.

The new visualization system makes it easier to identify connections between common metabolic pathways and biosynthetic pathways of interest, as described in the next section.

### Visualization of pathway results

The Hub Pathways Webserver (http://hpat.kavrakilab.org) was developed to execute user pathway searches using LPAT and HPAT. Once a search query has been completed, the user can view the pathway results in the webserver’s pathway visualization interface.

The visualization interface consists of a main panel to display the pathway search results and a fixed left panel for displaying information on the compounds and reactions contained in the pathway results, as well as filtering options that can be applied to the results. Pathway results are visualized as a single combined graph, where the nodes represent compounds and the edges represent enzymatic reactions. The starting compound node is highlighted in green and the target compound node is highlighted in red.

Each compound node can be clicked to display the common name and chemical structure of the compound at the top of the left panel. In addition to the visualization graph, the main panel displays the total number of pathways that are currently being visualized by the graph in the top left-hand corner. Note that for HPAT pathways, a pathway result containing precomputed hub path(s) is counted as one pathway when calculating the displayed total number of pathways, instead of counting all possible variations of the hub path(s) as separate pathways.

**Fig. 3:**
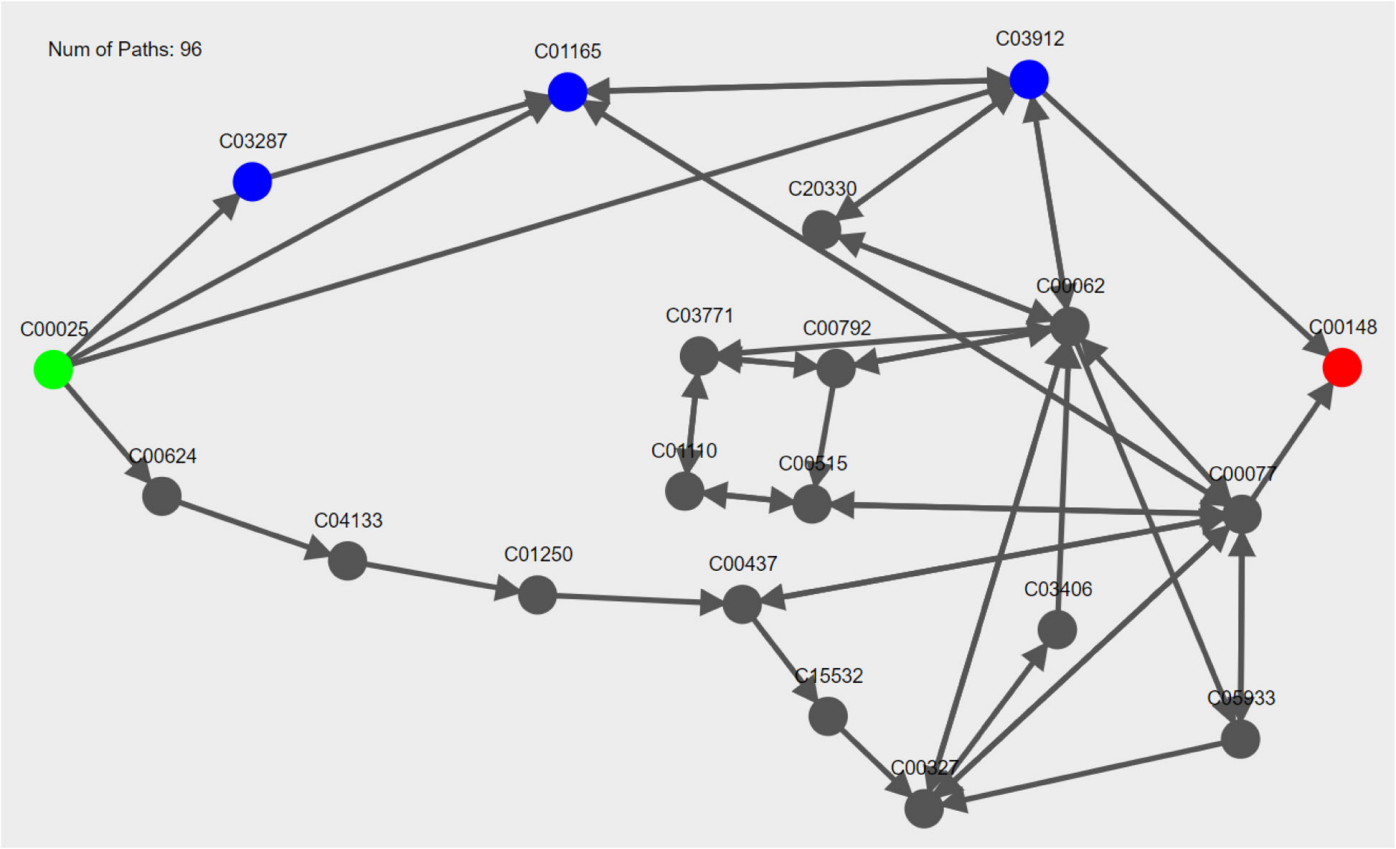
Visualization of LPAT pathway results from glutamate (C00025, green node) to proline (C00148, red node). Compounds in the canonical proline synthesis pathway are highlighted in blue. The arrows of the edges connecting the compound nodes indicate the directionality of the reactions, where double-sided arrows indicate the reaction is reversible. The total number of pathways (96 paths) in the graph is indicated in the upper left corner of the interface display

Combining all results into a single graph eliminates the redundancy of common path segments that appear across multiple pathway results, which provides a more condensed summary of result pathways compared to a list of individual pathways (see Fig. 3 as an example of how ninety six pathway results can be displayed in a single graph).

#### Hub pathway visualization

**Fig. 4:**
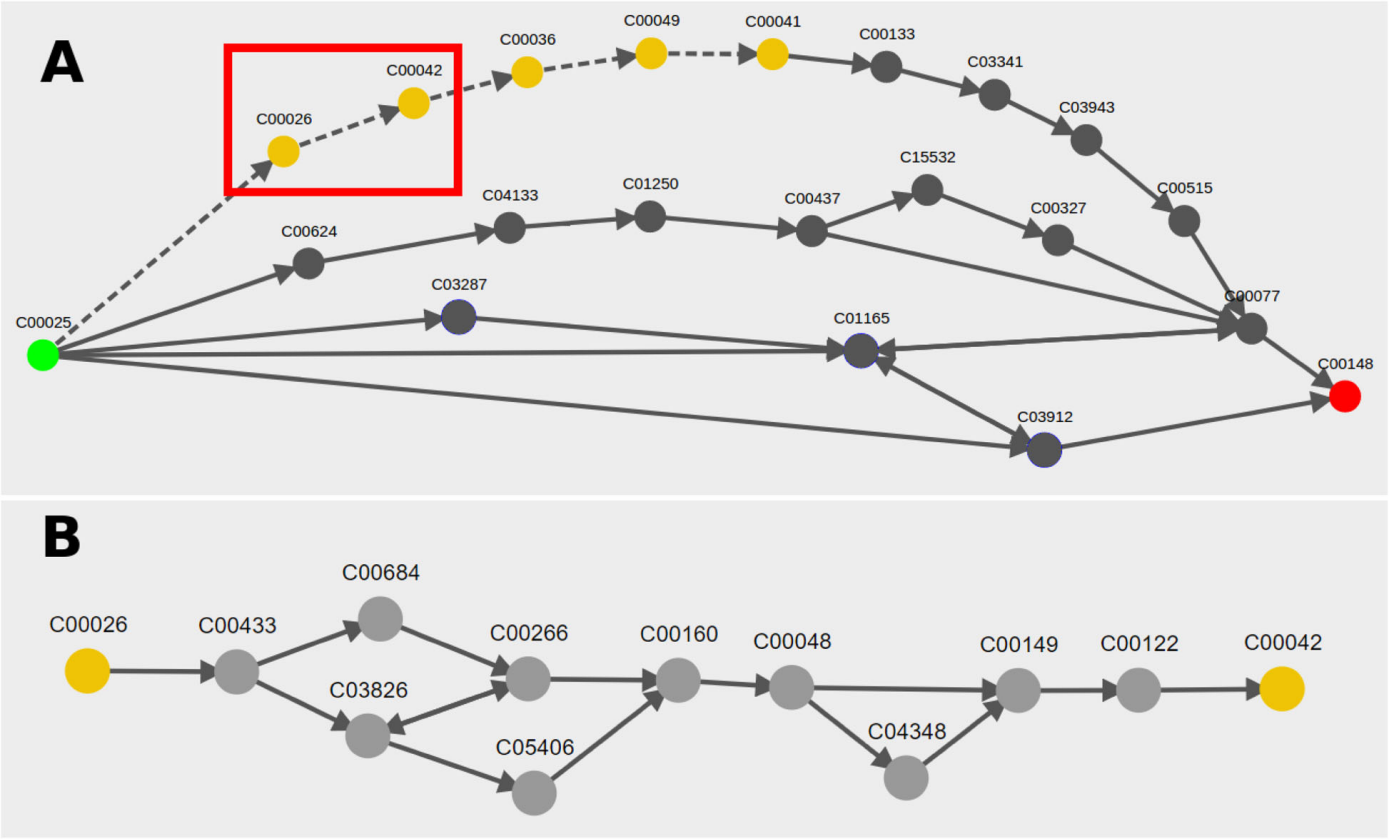
Visualization of pathway results for a hub search from glutamate (C00025) to proline (C00148). **a** The initial visualization abstracts the subpaths between several hub pairs as a dotted arrow, including the subpaths from 2-oxoglutarate (C00026) to succinate (C00042), highlighted by the red box. **b** Clicking on the dotted edge between C00026 and C00042 in view (**a**) displays all the subpaths between the hub pair as a single graph, where nodes represent compounds and edges represent enzymatic reactions

For HPAT results, the details of hub-to-hub pathways are abstracted such that there is a single dashed arrow to indicate that one or more subpaths exist between two hub compounds. Hiding the details of these common hub pathways simplifies the overall pathway view, allowing the user to get a clearer idea of which hub compounds are used as intermediates by the found pathways. Though all compounds and reactions between hub compounds are initially hidden from view, the user may click any dashed arrow between a pair of hubs to switch to a more detailed view of all the possible subpath connections between the hub pair (See Fig. 4).

### Filtering of pathways

**Fig. 5:**
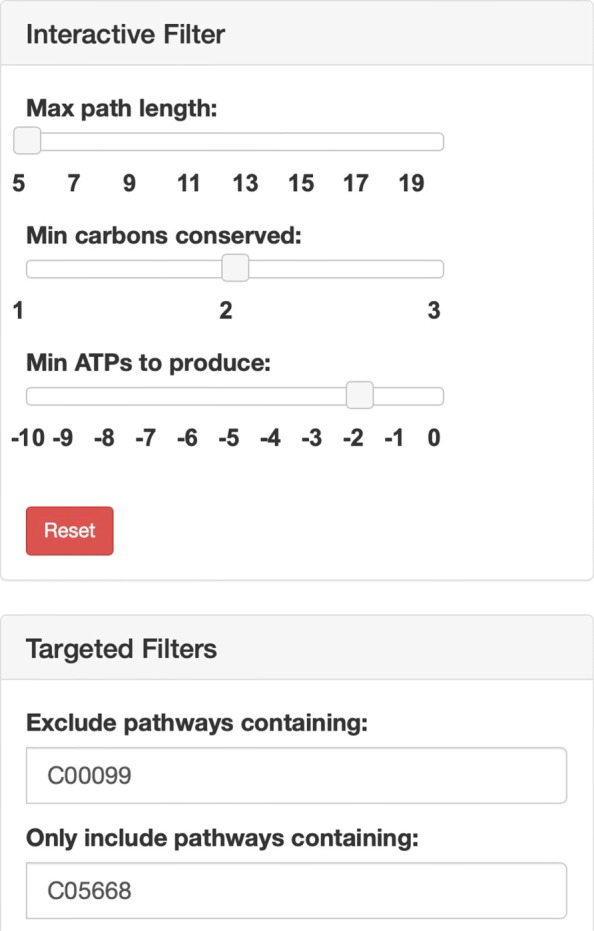
Filtering options for pathway results. A screenshot of filter options that can be found in the left column of the webserver’s pathway visualization interface. Users can (1) exclude pathways containing specific compounds and reactions by specifying their KEGG ids, (2) only include pathways containing specific compounds and reactions by specifying their KEGG ids, (3) exclude pathways with a path length longer than a given number of reactions, (4) exclude pathways that conserve less than a given number of carbon atoms, and (5) exclude pathways that produce less / consume more than a given number of ATPs

Since pathway searches may return tens of thousands of results, user are provided the option on the left panel of the visualization interface to filter results by compounds, path length, carbons conserved, and ATP usage (See Fig. 5). Users can also interact directly with the visualization graph by right clicking compounds to filter pathways containing the right-clicked compound. These filter options allow the user to quickly remove or highlight pathways, narrowing down the number of results the user must view at once.

#### Compounds

**Fig. 6:**
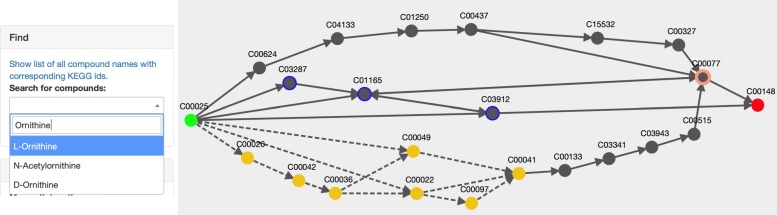
Highlighting ornithine in all pathway search results from glutamate to proline. For the pathway search from glutamate (C00025) to proline (C00148), if the user searches for “ornithine” in the “Find” selection bar, they will be presented a list of compounds that match their input. They can then select L-ornithine (C00077) from the “Find” compound selection list, and it will be highlighted with a bolded salmon-colored outline in the graph. Users can search for any compound in the graph by entering either the compound’s common name or KEGG id

Using the compounds filter, the user can specify compounds that they would like to exclude from or include in all displayed pathway results (Fig. 5). Users can also right click compound nodes in the visualization graph and choose to exclude or only include all pathways that contain that compound. To locate compounds in the visualization graph by their common names, the user can use the compound selection bar to highlight the compounds in the graph (Fig. 6).

**Fig. 7:**
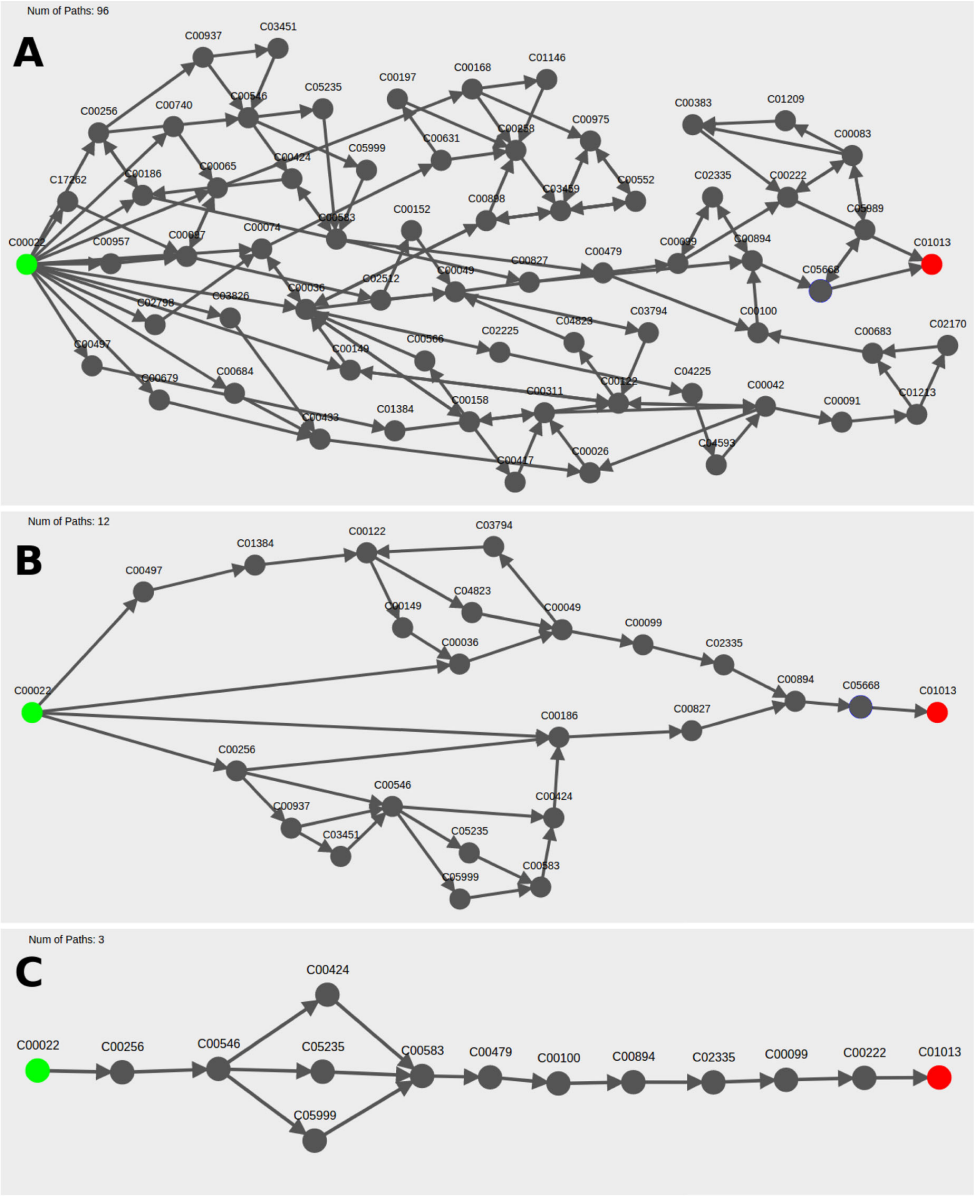
Filtering pathway results from pyruvate to 3-HP using compound selections. In the webserver visualization, users can specify which compounds they would like to include or exclude in pathway results by typing the KEGG compound ID(s) into the “Filter” text box or by right clicking the compound node(s) in the graph. For example, for **a** the LPAT pathway search results between pyruvate (C00022) and 3-HP (C01013), **b** pathways containing either 3-oxopropanoate (C00222) or propanoyl-CoA (C00100) can be excluded from the search, reducing the number of pathways displayed from ninety-six to twelve. **c** For the same pathway search, the user can require all pathways to contain both 3-oxopropanoate (C00222) and propanoyl-CoA (C00100), which reduces the number of pathways displayed from ninety-six to three

Novel pathways that were found by the search can be identified by excluding result pathways that contain specified compounds or by only including result pathways that go through specific compounds (See Fig. 7 for an example with 3-HP search results). This provides the user an option to quickly remove pathways with features that are not interesting to them without having to look at each pathway individually. The user can also switch between different compound filters without having to re-run the search.

#### Interactive sliders

In addition to the compound filter, the user also has access to three interactive sliders to filter pathways by (1) maximum path length, defined as the number of reactions included in the pathway, (2) minimum number of carbon atoms conserved between the start compound and the target compound, and (3) the maximum net number of ATP consumed throughout the pathway (Fig. 5). Path length and atom tracking have been used by several pathfinding approaches as a heuristic to find biologically feasible pathways. The default maximum path length is set to the minimum path length found in the results to prevent cases where an overwhelming number of pathways is initially displayed to the user. The default minimum carbons conserved is initially set to two carbons, since this filters out inefficient pathways that may break down the starting compound to single carbon compound intermediates (e.g., carbon dioxide) before building back up to the target compound.

Pathways that require less ATP (energy) tend to be more efficient and preferred in metabolic engineering, so the ability to filter pathways by ATP can narrow down the results based on energy efficiency. Unlike the previous two sliders, the ATP usage slider includes both positive and negative integers. Selecting a positive value *i*, filters out pathways that produce less than a net amount of *i* ATP molecules. Selecting a negative value −*i*, filters out pathways that consume more than a net amount of *i* ATP molecules. The default value for the ATP usage slider is −1, meaning that all pathways that consume more than a net amount of one ATP molecule will be filtered from the results.

**Fig. 8:**
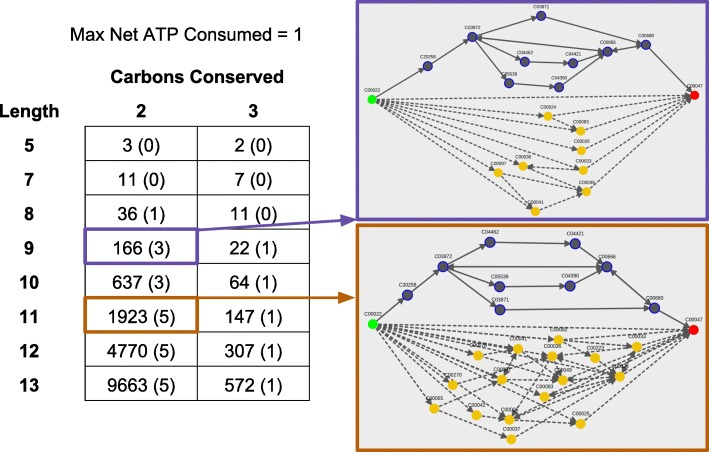
Filtering pathway results from pyruvate to lysine. The number of unique pathways found by HPAT from pyruvate to lysine for different combinations of maximum path length and minimum number of conserved carbons cutoffs (a maximum net ATP consumed of one is also applied for all combinations). The number of pathways includes all possible combinations of precomputed subpaths between hub pairs, and the number of known pathways that can be found using each filtering combination is reported in parentheses. All five known pyruvate synthesis pathways used as test cases in this paper are displayed when the max path length = 11 and minimum carbons conserved = 2. The visualization of the results for two different filtering combinations (length=9, carbons conserved=2, and length=11, carbons conserved=2) are shown on the right

Using the three sliders can significantly reduce the number of pathways displayed to the user. In some cases, the known pathways are captured at the extremes of the filters. For example, the known stachyose synthesis pathway can be recovered by sliding the path length filter to the minimum path length of three. However, aggressively filtering can exclude known pathways, such as the case for known lysine pathways (Fig. 8). This example illustrates that filters may need to be adjusted by the user to identify all interesting pathways. Using interactive filtering, users can view a manageable subset of pathways that match their criteria for identifying feasible pathways.

### Hub table size and selection

The results reported so far were produced using the hub table constructed using fifty hub compound selected by out-degree, listed as the 50 (O) hub table in Table 2. To ensure the hub table size and selection of hubs used for this study were reasonable and to observe how changes to the selected hub compounds affected pathway results, hub tables of varying sizes (20, 50, and 80 hubs) and varying hub selection criteria were comparatively evaluated (See Table 2). Three of the hub selection criteria tested were based on the compound’s connectivity in the metabolic network. Compounds could be selected by (1) largest in-degree, or how many reactions produced the given compound as a product, (2) largest out-degree, or how many reactions used the given compound as a reactant, or (3) largest sum of in-degree and out-degree. Two random hub selections of fifty compounds and the hub compounds selected by Araki et al. [43] were also included for the sake of comparison with the hub selections based on connectivity.

**Fig. 9:**
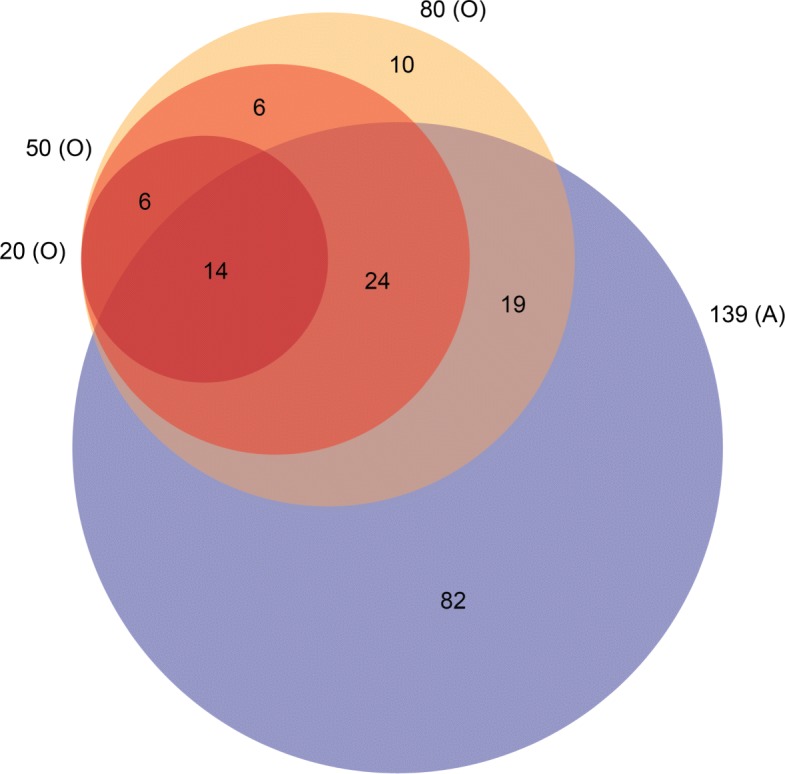
Overlap of hub compounds between the 20 (O), 50 (O), 80 (O), and 139 (**a**) hub tables. Though there are a few compounds contained in the out-degree hub table which are not included in the 139 (**a**) hub table, there is a significant overlap between the out-degree tables and the hub table constructed from the list of hub compounds taken from Araki et al. [43]. There are 68, 38, and 14 compounds shared between the 139 (**a**) hub table and the 80 (O), 50 (O), and 20 (O) hub tables respectively

**Fig. 10:**
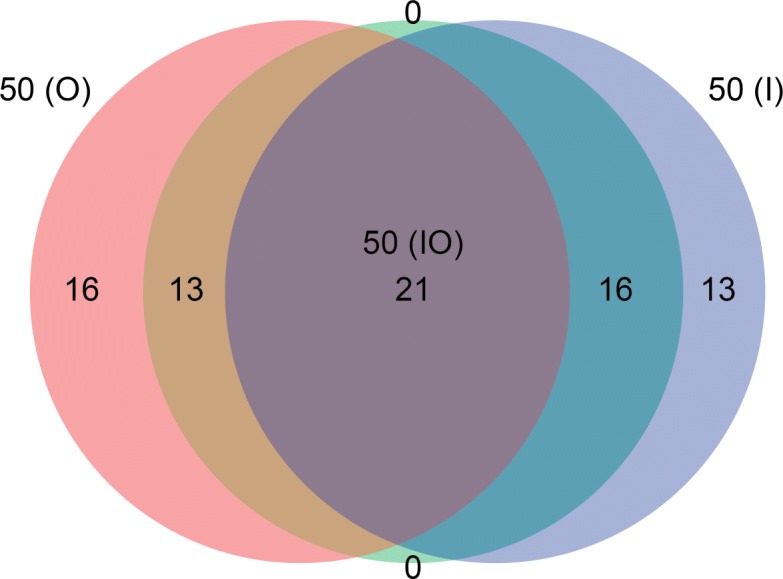
Overlap of hub compounds between the 50 (O), 50 (IO), and 50 (I) hub tables. The three hub tables share twenty one hub compounds. The hub compounds in the IO hub table are all encompassed by either the O or I tables. The O hub table has sixteen unique hub compounds and thirteen hub compounds shared with the IO table, but not the I table. The I hub table has thirteen unique hub compounds and sixteen hub compounds shared with the IO table but not the O table

The overlap of the different hub compound selections used to construct these hub tables are illustrated in Figs. 9 and 10. All hub tables had some overlap in hub compounds with the other tables. Thirty eight hub compounds are shared between the top 50 out-degree (O) hub table and 139 (A) hub table. The top 50 in-degree (I) hub table and the 50 (O) hub table share twenty one hub compounds. All hub compounds in the top 50 combined in-degree and out-degree (IO) hub table are contained within either the I table or O table and contains about the same number of hub compounds from both tables.

The two hub tables constructed from randomly selected hub compounds shared no overlap with each other. The 50 (R1) table has one hub (C00235) that is in the 50 (O) and 80 (O) tables and another hub (C00002) that is shared with the 139 (A) table. The 50 (R2) table has one hub (C00270) that is in the 50 (O), 80 (O), and 139 (A) tables. Otherwise, there were no other overlaps with the R1 and R2 tables.

After running hub searches using the 20, 50, and 80 (O) tables to find the canonical pathway test cases, the F_1_ scores of all the searches are relatively similar with the exception of three test cases: two (D-glucarate and 1,3-propanediol) where the search using the 80 (O) table fails to find the canonical pathway while the searches using the 20 and 50 (O) tables successfully identify the canonical pathway and one (3-HP, III) where search using the 80(O) table finds the known pathway and the searches using the 20 and 50 tables do not (See Table 3). Though it seems counter-intuitive that the larger 80 hub table fails to find a few canonical pathways that are identified by the smaller 20 and 50 hub tables, having a larger number of hub compounds increases the chances of the precomputed hub network being used during the online search, which can fail to find the known pathway due to not including the first or last hub compound of the known pathway as a hub closest to the start / target compound (see “Reasons for missing some canonical pathways” subsection in “Discussion” section for more details).

**Table 3:** F_1_ scores of hub tables of different sizes and hub selections for the known pathway test set, where an F_1_ score of one indicates that the HPAT search found the exact known pathway

	Number of Hubs							
	20 (O)	50 (O)	80 (O)	139 (A)	50 (IO)	50 (I)	50 (R1)	50 (R2)
**Pyruvate → Lysine I**	**1.00**	**1.00**	**1.00**	**1.00**	**1.00**	0.67	0.70	0.70
**Pyruvate → Lysine II**	**1.00**	**1.00**	**1.00**	**1.00**	**1.00**	0.67	0.70	0.70
**Pyruvate → Lysine III**	**1.00**	**1.00**	**1.00**	**1.00**	**1.00**	0.61	**1.00**	**1.00**
**Pyruvate → Lysine IV**	**1.00**	**1.00**	**1.00**	**1.00**	**1.00**	0.61	0.78	0.78
**Pyruvate → Lysine V**	**1.00**	**1.00**	**1.00**	**1.00**	**1.00**	0.10	0.30	0.30
**Glutamate → Proline**	**1.00**	**1.00**	**1.00**	**1.00**	**1.00**	**1.00**	**1.00**	**1.00**
**UDP-Galactose → Stachyose**	**1.00**	**1.00**	**1.00**	**1.00**	**1.00**	**1.00**	**1.00**	**1.00**
**alpha-D-glucose → Phenylpyruvate**	0.71	0.71	0.71	0.62	0.74	0.71	0.71	0.71
**alpha-D-glucose → Dopamine**	0.76	0.59	0.59	0.60	0.65	0.76	0.76	0.76
**Pyruvate → 3-HP, I**	**1.00**	**1.00**	**1.00**	**1.00**	**1.00**	**1.00**	**1.00**	**1.00**
**Pyruvate → 3-HP, II**	0.38	0.50	0.50	0.50	0.50	0.60	0.33	0.33
**Pyruvate → 3-HP, III**	0.55	0.60	**1.00**	**1.00**	0.60	**1.00**	0.78	0.78
**Pyruvate → 3-HP, IV**	0.38	0.42	0.50	0.50	0.43	**1.00**	0.71	0.71
**Pyruvate → 3-HP, V**	0.40	0.36	0.36	0.36	0.36	0.36	0.40	0.40
**Pyruvate → 3-HP, VI**	0.36	0.36	0.18	0.18	0.36	0.67	0.36	0.36
**Pyruvate → 3-HP, VII**	0.36	0.53	0.77	0.77	0.53	0.77	0.50	0.50
**Glucose → Glucaric acid**	**1.00**	**1.00**	0.38	0.44	0.21	**1.00**	**1.00**	**1.00**
**alpha-D-glucose → 1,3-propanediol**	**1.00**	**1.00**	0.57	0.89	**1.00**	**1.00**	**1.00**	**1.00**
**Tryptophan → Melatonin**	**1.00**	**1.00**	**1.00**	**1.00**	**1.00**	**1.00**	**1.00**	**1.00**

The closer the F_1_ score is to one, the more similar the found pathway’s reactions are to that of the canonical pathway. The different suffixes in column names indicate how hubs were selected: IO, sum of in- and out-degree; A, from Araki et al. 2015; O, out-degree; I, in-degree; R*n*, randomly

The search time typically ranged from seconds to a few minutes across all hub tables. Both the median and average total search time was lowest for the 80 (O) hub table and highest for 20 (O) hub table (see Additional file 8). When looking at only the hub-to-hub search time, the time increases with the hub table size (see Additional file 9). However, increasing the hub table size also increases the likelihood of the start compound being a hub compound, which reduces the search time in two ways: (1) the start to hub search is not required, since the start compound is already in the hub table and (2) starting with a hub compound significantly reduces the search time for the hub to target compound, since there are fewer variations of carbons conserved. Because of this, the search time, on average, is less for the largest hub table than the mid-sized and small hub tables.

The 20, 50, and 80 (O) hub tables were also compared based on their coverage of reactions present in KEGG pathway modules, a curated list of pathways featured by KEGG as central to metabolism across organisms. A reaction in a KEGG pathway module is considered “covered” by a hub table if the reaction is present in at least one precomputed subpath stored in the hub table. An increasing number of reactions across KEGG modules are covered as the size of the hub table increases; however, the increase between the 20 and 50 hub table is larger than between the 50 and 80 hub table (see Table 4 for more details). These trends remained the same when examining the reaction coverage of the forty eight KEGG pathway modules that specifically correspond to the reaction modules found across many known pathways identified by [29] (see Additional file 7). This suggest that though increasing the size of the hub table does increase the number of KEGG module reactions contained in the hub table’s precomputed subpaths, there are diminishing returns for increasing the number of hub compounds. In the end, the hub table constructed with 50 hubs was chosen for this study, since the 50 hub table both covered a more sizable number of reactions in the KEGG modules than the 20 hub table while also enabling the hub search to find more canonical pathways than the 80 hub table.

**Table 4:** The percentage of the 310 KEGG pathway modules that had all of their reactions, at least half of their reaction, and none of their reactions contained by hub tables constructed with varying numbers and selections of hub compounds

	Different hub tables							
	20 (O)	50 (O)	80 (O)	139 (A)	50 (IO)	50 (I)	50 (R1)	50 (R2)
All	19.35	26.77	32.58	34.52	24.84	28.71	13.23	20.00
At least half	42.90	54.19	61.61	63.55	53.23	56.13	35.16	45.48
None	37.42	26.13	23.55	18.06	31.29	30.00	42.58	35.16

The KEGG pathway modules represents well-documented and important metabolic pathways that ideally should be covered by the precomputed subpaths stored in hub tables. The different suffixes in column names indicate how hubs were selected: IO, sum of in- and out-degree; A, from Araki et al. 2015; O, out-degree; I, in-degree; R*n*, randomly

Similar to the different hub table sizes, the three hub selections (O, I, and IO) described in Table 2 were compared based on finding the canonical pathway test cases. The size of the hub tables were all fixed at 50 hub compounds. The search using the 50 (O) hub table was able to find the canonical pathways for eleven out of nineteen test cases, whereas the search using the 50 (I) and the 50 (IO) tables found eight and ten canonical pathways respectively (Table 3). The differences between these three hub selections were due to the start and/or target compound being included in the hub compounds list for one hub selection but not included for the others. As a result, any failure to identify the closest hub compounds to the start or target compound (which is the first step in the hub search, see Fig. 1c and the “First and last hubs selection” subsection in “Methods” section for more details) would unequally impact the F_1_ scores. For example, both the start and target compound in the lysine pathways were hub compounds for the 50 (O) and 50 (IO) hub tables, but not the 50 (I) table. The hub search meta-algorithm did not identify the correct closest hub for the search using the 50 (I) table, resulting in the search not finding the canonical pathways. Meanwhile, since the start and target compound were hubs for the 50 (O) and 50 (IO) tables, the canonical pathways were able to be found in these cases.

All three of the hub selections had comparable coverage of the KEGG module reactions and had higher coverage than that of the hub tables constructed using randomly selected hub compounds when looking at all 310 KEGG pathway modules (see Table 4). However, when looking at only the KEGG pathway modules that correspond to reaction modules identified by [29], the out-degree hub selection covered more KEGG module reactions than the in-degree and the total degree selections (see Additional file 7). Search times for the hub tables varied for each test case and in almost all cases range from a few seconds to a few minutes. None of the three curated hub selections resulted in a significantly lower total search time compared to the others across all the pathway test cases (Additional file 8). For this study, the 50 (O) hub table was used since this table enabled the search to find the most canonical pathways.

The 139 (A) hub table constructed based on the hub compounds in Araki et al. [43] had the highest coverage of KEGG modules. Though the 139 (A) table showed significantly wider coverage of the KEGG pathway modules corresponding to reaction modules (85.42% of these KEGG modules were at least partially covered by the 139 (A) table while all the other hub tables only partially covered 42–44%), in most cases the coverage of the 139 (A) table was not significantly different from the 80 (O) table.

The number of pathways found for each pathway test case by the hub search using the different hub tables are in Additional file 10. The number of pathways are recorded for no hub / single hub search (Fig. 1a and b), and the start to hub, hub to hub, and hub to target components of the hub search (Fig. 1c). Both the smaller hub tables (i.e., 20 (O)) and the hub tables constructed using randomly selected hubs tend to have a larger number of no hub / single hub pathways and a smaller number of hub to hub pathways than the larger hub tables (i.e., 80 (O) and 139 (A)), illustrating that the hub to hub search is more actively used when the hub tables contain a large number of highly-connected hubs.

## Discussion

This paper outlines the first steps to taking advantage of repeated, common subpaths in the metabolic network and utilizing these subpath modules to improve the organization of pathway results identified by metabolic pathfinding approaches. HPAT was shown to find more known pathways than LPAT by using a diverse collection of precomputed subpaths. The capability of HPAT to find novel pathways was illustrated through the alternative 3-HP pathways identified by the search. The paper also introduces a new way to visualize pathway results as a single interactive graph, where subpaths between hub compounds can be expanded or hidden from view to simply visualization. Filtering tools enable users to quickly sift through pathway results without having to click individually through a list of pathways or rely on a pre-determined ranking heuristic to present the best results.

### Reasons for missing some canonical pathways

Though the HPAT meta-algorithm recovered many of the known pathways in the test set, a few known pathways were not found due to a failure of the HPAT meta-algorithm to identify the correct first or last hub in the pathway. The identification of closest hubs is based on the assumption that the hub compounds with the highest chemical similarity score to the start and target compounds would be the most likely hub compounds included in the pathway result (See subsection “First and last hubs selection” subsection in “Methods” section).

As seen in the results, the structure of the first or last hub compound may not necessarily resemble the start or the target compound respectively due to (1) an addition of a coenzyme or large structural addition along the way (i.e. coenzyme-A) or (2) the hub compound present in the known pathway is many reactions away from both the start and the target compound. In these cases, the heuristic is unable to identify the correct closest hub compound.

One alternative method is to directly identify the hub compounds that are the least reactions away from the start and the target, using a breadth first search. Though it would take more time to compute, this approach would be able to find closest hubs with chemical structures that differ significantly from the start and target compounds.

Another promising option is to use the chemical similarity measure to identify a recommended set of closest hub pathways, then provide the user the option to add or remove hub compounds to the recommended set before performing the hub search. This allows the user to provide expert guidance on which closest hubs should be explored in the search, while also presenting the user with a smaller subset of recommended hub compounds so that the user does not need to examine all possible hub compounds.

### Conservation of carbon atoms

All the metabolic pathfinding experiments described in this paper had the constraint of conserving at least two carbon atoms from the start compound to the target compound. When using the term ‘conservation’ we are specifically referring to retaining a subset of carbon atoms present in the starting compound through each biochemical transformation in the pathway through to the final target compound. Conserving some minimum number of carbon atoms throughout the pathway is done to ensure that the start compound is being utilized in production of the target compound, and this heuristic has been used by several existing metabolic pathfinding methods [9, 10, 12, 17, 19, 44, 45]. However, if the number of atoms conserved throughout the pathway is relatively small compared to the number of atoms in the start compound or target compound, the carbon conservation heuristic may not be as effective at preventing the search from finding infeasible pathways, since the search is still able to find pathways that break down the start compound to a much smaller compound (i.e., CO_2_) then build that compound back up to produce the target compound.

There are a few ways to prevent the search from including unnecessary build up / break down steps in the pathway results. First, the user of the hub pathway search has the choice to maximize the number of carbons conserved throughout the pathway, so that all pathways found by the hub pathway search conserve the largest possible numbers of carbon atoms from start to target compound. Another option that can be easily made available is for the user to specify what percentage of carbon atoms that exist in the start compound or target compound should be conserved throughout the pathway. This way, a larger number of carbon atoms are conserved when the start and target compounds are large, preventing the search from finding pathways with unnecessary break down / build up steps, while also accounting for scenarios where the start compound or target compound is small, and conserving only one or two carbon atoms will not cause the search algorithm to find lengthy infeasible pathways.

### Hub compound selection

The selection of hub compounds used in the hub table can have a significant impact on how frequently the hub table is utilized in the search. When hub compounds were selected randomly, the hub table was often not used in finding the canonical pathways since no feasible pathways passed through the precomputed paths. Meanwhile, when the hub compounds were selected by their degree of connectivity with other compounds, the hub table was used for finding more than half the canonical pathways. Increasing the number of hubs also increases the use of the hub table in searches, which in some cases, had a negative effect on the F_1_ score when the chemical similarity heuristic did not identify the correct first or last hub compound. As a result, increasing the hub table size did not result in better F_1_ scores for finding canonical pathways even though larger hub tables had more coverage of the KEGG modules and KEGG reactions than smaller hub tables.

### Improvements to visualization

Metabolic pathfinding has many potential applications, from suggesting new pathways for metabolic engineering to help with understanding the metabolic space in multi-species communities. It is essential for these tools to be flexible and interactive, so users can quickly find the information that they are seeking. To this end, better organization and visualization of predicted pathways will enable further development and utilization of the available, rapidly growing metabolic information.

The sliding filters in the web server visualization allow for more flexible user interaction with the pathway search results; however, there are several improvements that could be made for an easier user experience. For example, the layout of the pathways can be intimidating to users, especially for large pathway graphs which often resemble a hairball of nodes and edges. One solution to visualizing a complex, disorganized network of predicated pathways is to utilize an existing scaffold for organization. This could be accomplished by overlaying the predicted pathways on curated representations of global metabolic networks, such as those from KEGG, which have been designed to minimize overlapping paths and to facilitate pathway navigation. Alternatively, introducing more hierarchical elements described in [31] could further condense the graph while still giving the user a feel of the connectivity and scope of the network.

More interactive filters based on other common search heuristics can be added to the webserver to allow the user to narrow the search. For example, an organism-based filter could allow users to view pathways that only contain enzymatic reactions from specific organisms of interest. A filter based on thermodynamic (*Δ*G) cutoffs could also be included for users to remove pathways that contain enzymatic reactions with unfavorable thermodynamics.

### Standardization and Integration

The Hub Pathways Webserver is currently a stand-alone tool, to gather feedback from the community. Such feedback and the availability of the code will drive further in-depth development. Ultimately, the development of an open-source suite of metabolic pathway visualization tools will help insure the sustainability and continued usage of these visualization tools over the years. In the future, it would be ideal for the Hub Pathways Webserver to be modulized such that the webserver’s functionalities can be seamlessly integrated with and supported by existing, well established open-source network visualization softwares like Escher [46] and Cytoscape [47]. Utilizing common standardized formats for compounds and reactions (i.e., SBML, MDL mol, and MDL rxn files) as inputs to the webserver could also bolster the webserver’s utility to the community. Realizing these changes is an ongoing process, and feedback and collaboration from the community is encouraged to achieve a more integrated and standardized environment for visualizing novel pathways.

### Future directions

The current hub search method only explores linear pathways, which tracks the conversion of a single compound throughout the pathway. However, most metabolic pathways are more accurately represented as branching paths and require multiple intermediate compounds at each enzymatic reaction step. The easiest way to introduce branched pathways into the hub search is to identify potential merge points between subpaths connecting a pair of hubs, similar to how the BPAT-M algorithm [48] finds branched pathways by merging linear pathways with the same start and target compound. Identifying potential branch pathways that help to feed by-product compounds back into the main pathway can result in a significant improvements in production. This is illustrated in a recent study by South et al. [49], where crops were modified to have an alternate metabolic pathway that more efficiently recycled unproductive side intermediates of photosynthesis and subsequently showed a 40% increase in biomass in the field.

In this paper, the metabolic pathfinding problem is framed as a graph-search problem similar to LPAT and BPAT [9]. However, there are other well-studied methods for identifying metabolic pathways, including constraint-based optimization methods. In order to better compare the performance of the hub search method with existing optimization approaches, the next step is to frame the hub pathway search approach presented in this paper as an optimization problem. This step could also facilitate the incorporation of more advanced properties such as taking into account steady state mass balance constraints.

Beyond its applications in metabolic pathfinding, the set of hub compounds identified in this study could be used as a tool to compare and describe the structure and properties of metabolic networks from different organisms or communities. For this paper, all compounds and reactions in the KEGG database were included in the searched metabolic network. However, there are cases where only a subset of the available reactions (i.e., only reactions from a specific species or community of organisms) are of interest to the user. The complexity and properties of the metabolic network composed of this subset may significantly differ from the entire known metabolome and other subsets of the metabolic network. Examining how the selection of hub compounds differ for a range of organisms, and which hub compounds are ubiquitous across all species, could lead to more insights about the roles these compounds play in metabolism. Also, assuming that the precomputed paths between hub compounds are a good representation of common metabolic modules across species, identifying which organism(s) contain the most of these precomputed subpaths could be a way to identify potential chassis organisms for metabolic engineering. This study’s initial exploration on how to select hub compounds and construct precomputed hub tables containing common metabolic modules serves as a good starting point for examining different organisms and communities in the future.

## Conclusion

The HPAT meta-algorithm takes advantage of the modularity of metabolic pathways by precomputing short subpaths (containing no more than ten reactions) that conserve carbon atoms between common hub compounds, and organizing these subpaths as a hub table for quick lookup during the graph-based search. The HPAT meta-algorithm was able to find a variety of known pathways and demonstrated its capability to find novel pathways. The interactive visualization of result pathways by the Hub Pathways Webserver enables users to filter pathways using intuitive heuristics, giving them more control over which pathways they see. Users also gain the flexibility to view a wide range of pathways without having to decide on cutoff parameters before the search.

## Methods

The hub pathway search meta-algorithm with atom tracking (HPAT) uses a graph-based atom tracking search approach for finding metabolic pathways. HPAT consists of an offline component, where paths between hub compounds are precomputed and stored in a hub table, and an online component, where the hub table is utilized for pathway search. Subpaths between hub compounds are assumed to appear frequently in pathway search results, since hub compounds are the substrates or products of a large number of enzymatic reactions. By precomputing and storing these subpaths, pathways between any pair of hub compounds can be easily explored and added to a pathway search. These precomputed subpathways are indexed by both the hub pair and the uniquely conserved carbon atom tuples for faster look-up.

### Offline component: precomputation of hub pathways

**Fig. 11:**
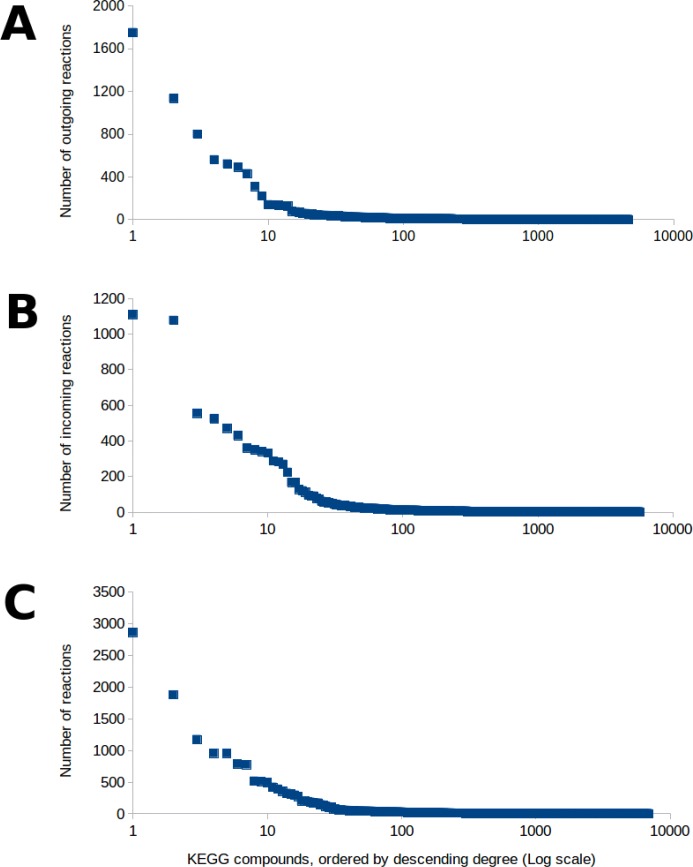
The number of reactions each compound in KEGG participates in as **a** a reactant (out-degree, used for O tables), **b** a product (in-degree, used for I tables), or **c** as a reactant or a product (total degree, used for IO tables). As illustrated in all three of the graphs, there are a few compounds that are involved in a large number of reactions, whereas the majority of compounds take part in only a handful of reactions

To construct the hub table, hub compounds must first be selected from the pool of known metabolites. Hub compounds are defined as key metabolites found across multiple pathways, such that we would expect many pathways to contain subpaths between two or more hub compounds. If the metabolic network is represented as a bipartite graph of compound nodes and reaction nodes, hub compounds can be identified as compound nodes connected with many reaction nodes. Three hub selections based on the in-degree, out-degree, and combined degree of compounds were used to construct the O, I, and IO hub tables described in the “Results” section. These hub selections were determined by ranking each KEGG compound by the number of reactions that contained the compound as a reactant and/or product (see Fig. 11), then selecting the compounds that were involved in the largest number of reactions.

Before hub compounds were selected, a set of KEGG compounds that would not make good hub compounds were removed from consideration based on a list of pre-determined rules (see Additional file 2). These removed compounds include currency metabolites and side compounds that are used to provide energy or to balance the reaction but do not contribute any carbon atoms to the target compound.

### Hub table construction

The hub table is constructed by running a linear pathway search between each possible pair of hubs using the Linear Pathfinding with Atom Tracking algorithm (LPAT) from Heath et al. [9]. All precomputed subpaths were required to conserve at least two carbon atoms (or at least one carbon atom, if the start or end hub compound contain only one carbon). To limit the size of the hub table, all paths stored in the hub table are ten or less reactions long, and a conditional maximum of one hundred subpaths are stored per hub pair. The maximum of one hundred subpaths may be exceeded only if all subpaths stored in the hub table conserve a unique set of carbon atoms, to ensure that all possible variations of conserved carbon sets are present in the hub table.

In addition to the start and target compound, LPAT also takes several adjustable input parameters, including the minimum number of carbon atoms to conserve, the number of shortest pathways to return (K), the maximum search depth, and whether or not to consider all reactions as reversible. The values used for these parameters can be found in Additional file 3. Although LPAT is used in this study, the HPAT meta-algorithm can be generalized to use any linear pathfinding method with atom tracking to find precomputed pathways.

**Fig. 12:**
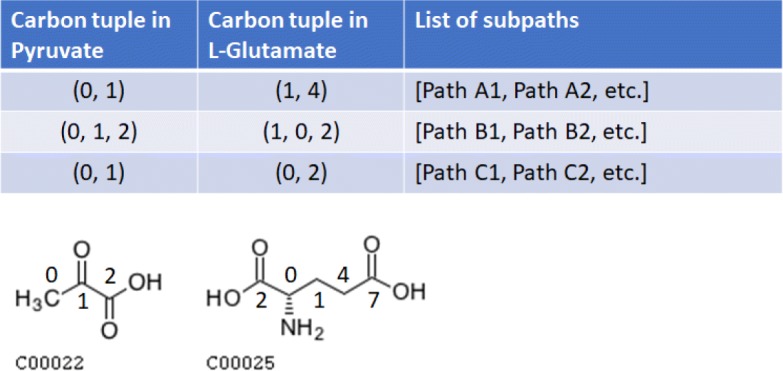
The organization of the hub table for a pair of hubs. Hub 1 is the compound to the bottom left and hub 2 is the compound to the bottom right. Each hub compound has numbered carbon atoms corresponding to the mappings displayed in the hub table. Individual rows in the table represent a unique tuple of carbons conserved between hub 1 and hub 2, where the carbon numbers of hub 1 in the first column correspond with the carbon numbers of hub 2 in the second column. The third column contains all the subpaths between hub 1 and hub 2 that are grouped under this tuple of conserved carbons

All precomputed subpaths between hub compounds are stored in the hub table. The subpaths are indexed first by hub pair and then by the tuples of conserved carbons from the start hub to the end hub (Fig. 12), which were tracked by the LPAT algorithm. Indexing subpaths by hub pair enables the HPAT meta-algorithm to quickly access all possible subpaths given a start hub and an end hub. The subpaths are further organized by tuples of carbon atoms conserved so the HPAT meta-algorithm can look up subpaths that conserve the required number of carbon atoms during the online search step. Indexing subpaths by tuples of carbons conserved can condense a list of a hundred subpaths to two or three groups of subpaths. For this study, the precomputation and organization of subpaths for all hub tables were performed on a Rice cluster, with each hub table taking a few hours to compute.

### Online component: pathway search

The online component of the hub search draws on both the linear pathway search and the precomputed hub table. The hub search separately explores three types of pathways: (1) pathways containing no hub compounds, (2) pathways containing one hub compound, and (3) pathways containing at least two hub compounds (See Fig. 1 in “Results” section). These pathways are found separately because for finding pathways containing one or no hub compounds, the precomputed network is not used for the search, while for finding pathways containing two or more hubs, the precomputed network is used.

A linear pathway search which excludes all hubs in the network is first run to find pathways with no hubs. Second, linear pathway searches which exclude all hubs except a single hub compound are run to find pathways with only one hub. The single hub searches are limited to only the *n* hubs that are closest to the start compound or to the target compound based on chemical similarity. Similar to the hub table construction step, the hub pathway search takes several input parameters to adjust the breadth of the search, including the maximum number of hubs in a hub pathway and the maximum number of reactions in a hub to hub path. The values used for the input parameters are described in the Additional file 4.

### First and last hubs selection

Start and end hub compounds were identified using the KEGG SIMCOMP similarity measure [50]. SIMCOMP is a graph-based method for scoring the structural similarity between compounds. The compound structure is represented by a graph with atoms represented as nodes and bonds represented as edges. SIMCOMP identifies the maximal cliques between compound graphs by comparing either the atom nodes or the bond edges, then calculating the similarity score as the number of nodes or edges matched between the compound graphs divided by the total number of nodes or edges present in the smaller compound. SIMCOMP also incorporates biochemical features (i.e., chirality) into the final similarity score. We assume that hub compounds that are structurally similar to the start compound or the target compound are more likely to be part of a feasible pathway connecting the two compounds. SIMCOMP was used to rank the similarity of all hub compounds to the start compound of the search, as well as the target compound. In this study, the three hub compounds with the top SIMCOMP similarity scores to the start compound were selected as the first hubs, and likewise, the three hub compounds with the top SIMCOMP similarity scores to the target compounds were selected as the last hubs in the search. By limiting the search to the top three scoring start hubs and last hubs, we can reduce the scope of the search to completely explore all possible paths going through the selected first hubs and last hubs instead of having to exhaustively search through all combinations of hubs. However, the number of closest hub compounds can be raised to obtain more diverse result pathways.

## Supplementary information

**Additional file 1** The test set of nine unique pairs of start and target compounds. The “num. carbons” columns report the number of carbon atoms in the start and target compounds. The “known paths” column refers to the number of canonical pathways found in KEGG or in other literature. The “path length” column refers to the number of reactions in the canonical pathway(s).

**Additional file 2** A list of all the exclusion rules used to curate the hub compound list. The left column provides the rule description, while the right column provides a list of compounds (with KEGG ids) that were removed due to the corresponding rule.

**Additional file 3** Hub table construction parameters. Values of the input parameters used for precomputing subpaths for all the hub tables in this paper.

**Additional file 4** Hub pathway search parameters. Values of the input parameters used for the hub search. These values can be adjusted to increase the number and diversity of pathways found.

**Additional file 5** LPAT search parameters. Values of the input parameters used for the LPAT search as a comparison to HPAT. These values were chosen to be comparable to the parameters used in the HPAT search.

**Additional file 6** An illustration of which steps of hub search (as shown in Figure 1) would find the canonical pathway for each of the pathway test cases using the different hub tables. For combinations marked with an “A,” the canonical pathway is found by the no-hub search. For combinations marked with a “B,” the single hub search is required to find the canonical pathway, and for combinations marked with a “C,” the canonical pathway would be found by the hub-to-hub search. Combinations that are also marked with a * indicate that the hub search failed to find the canonical pathway in this case. Hub tables constructed with more hubs must more frequently use the hub to hub search to find canonical pathways, while the hub tables constructed with a random selection of hubs do not use the hub to hub search to find the canonical pathways. The different suffixes in column names indicate how hubs were selected: IO, sum of in- and out-degree; A, from Araki et al. 2015; O, out-degree; I, in-degree; R*n*, randomly.

**Additional file 7** The percentage of the forty eight KEGG pathway modules corresponding to the reaction modules identified by [29] that had all of their reactions, at least half of their reaction, and none of their reactions contained by hub tables constructed with varying numbers and selections of hub compounds. The different suffixes in column names indicate how hubs were selected: IO, sum of in- and out-degree; A, from Araki et al. 2015; O, out-degree; I, in-degree; R*n*, randomly.

**Additional file 8** Total time of searches using hub tables of different sizes and hub selections for the known pathway test set, run on an Intel Core i7-4790 with 16 GB RAM. The different suffixes in column names indicate how hubs were selected: IO, sum of in- and out-degree; A, from Araki et al. 2015; O, out-degree; I, in-degree; R*n*, randomly.

**Additional file 9** Times for the hub-to-hub part of the hub search, using hub tables of different sizes and hub selections for the known pathway test set. The different suffixes in column names indicate how hubs were selected: IO, sum of in- and out-degree; A, from Araki et al. 2015; O, out-degree; I, in-degree; R*n*, randomly.

**Additional file 10** Number of pathways found for the (1) no hub / single hub part of the search and (2) the hub-to-hub part of the search, using hub tables of different sizes and hub selections for 19 pathway test set. For the hub-to-hub pathways, unique combinations of hub pairs that conserve different sets of carbons are counted as individual pathways; however, the different combinations of subpaths between hub pairs are not counted as separate pathways. For example, given hub compounds H1, H2, and H3, (1) H1 →H2 and H1 →H3 are counted as separate pathways, (2) H1 →H2 conserving two carbon atoms and H1 →H2 conserving a different set of two carbon atoms are counted as separate pathways, but (3) any subpath variations between H1 and H2 (i.e., H1 →N1 →H2 and H1 →N2 →H2, where N1 and N2 are non-hub compounds) are not counted as separate pathways, given that they conserve the same carbon atoms from H1 to H2. The different suffixes in column names indicate how hubs were selected: IO, sum of in- and out-degree; A, from Araki et al. 2015; O, out-degree; I, in-degree.

## Data Availability

The datasets used and/or analysed during the current study are available from the corresponding author on reasonable request.

## Abbreviations

3-HP: 3-hydroxypropanoate
ATP: Adenosine triphosphate
HPAT: Hub pathway search with atom tracking
KEGG: Kyoto encyclopedia of genes and genomes
LPAT: Linear pathway search with atom tracking
